# Methanol Mitigation in Kiwifruit Wine Fermentation Through Process Regulation: Effects on Physicochemical Properties and Aroma Characteristics

**DOI:** 10.3390/foods15173150

**Published:** 2026-09-05

**Authors:** Weihao Chen, Ju Shen, Jiaxin Xu, Yilin You, Weidong Huang, Jicheng Zhan

**Affiliations:** College of Food Science and Nutritional Engineering, China Agricultural University, Beijing 100083, China

**Keywords:** methanol, kiwifruit wine, pectinase, fermentation temperature, clarification centrifugation, orthogonal design

## Abstract

Methanol formation is a critical safety concern in kiwifruit wine due to the high pectin content of kiwifruit. This study aimed to establish an effective strategy to reduce methanol formation while maintaining the fermentation performance and sensory quality of kiwifruit wine. The effects of fermentation temperature, pectinase dosage, and clarification centrifugation conditions on methanol production were investigated, and the selected process-regulation strategy was further evaluated by analyzing physicochemical properties, volatile aroma compounds, and sensory characteristics. The results showed that pectinase dosage was the predominant factor affecting methanol formation. Under the selected process conditions identified within the tested parameter ranges (fermentation at 19 °C, pectinase dosage of 0.02 g/L, and centrifugation at 2500 r/min), the methanol content decreased to 167.45 ± 2.20 mg/L, representing a 37.39% reduction compared with the control group, while the ethanol content remained at 12.66 ± 0.04% (*v*/*v*). Moreover, the wine exhibited favorable physicochemical characteristics, including improved retention of phenolic compounds and balanced organic acid composition. Aroma profiling using GC–MS and multivariate statistical analysis demonstrated that the optimized wine exhibited a volatile aroma pattern comparable to that of the control wine, indicating that methanol mitigation did not substantially impair the characteristic flavor of kiwifruit wine. Sensory evaluation further confirmed the improved overall acceptability of the processed product. This study provides a practical approach to producing safer kiwifruit wine while preserving quality attributes.

## 1. Introduction

Kiwifruit wine has attracted increasing interest as a value-added fermented fruit product because of its characteristic acidity, nutritional components, and distinctive aroma [1,2]. The presence of at least 14 commercially available kiwifruit alcoholic beverages in China, together with reported consumer acceptance of 67% and purchase intention of 39% for a kiwifruit-based fermented beverage, suggests promising market potential for this product category [3,4]. Kiwifruit is rich in soluble sugars, organic acids, vitamin C, polyphenols, dietary fiber, and aroma precursors, which provide a suitable basis for fruit wine fermentation [5]. A recent study comparing wines produced from 12 red-, yellow-, and green-fleshed kiwifruit cultivars reported pH values of 3.16–4.21, total soluble solids of 6.47–9.00 °Brix, titratable acidity of 9.60–19.48 g/L, and residual total sugars of 3.38–9.73 g/L. These wines also retained considerable amounts of bioactive compounds, including vitamin C, total polyphenols, and flavonoids, which further contributed to their nutritional and functional characteristics [6]. In China, kiwifruit wine is usually produced from fresh kiwifruit pulp through enzymatic treatment, sugar and acid adjustment, alcoholic fermentation, clarification, and storage [7,8,9]. Cultivars such as ‘Xu Xiang’, ‘Hayward’, and other yellow- or green-fleshed kiwifruit have been used for wine production, and kiwifruit has also been blended with pear, Cornus officinalis, and other fruits to improve flavor complexity [10].

**Figure 1:**
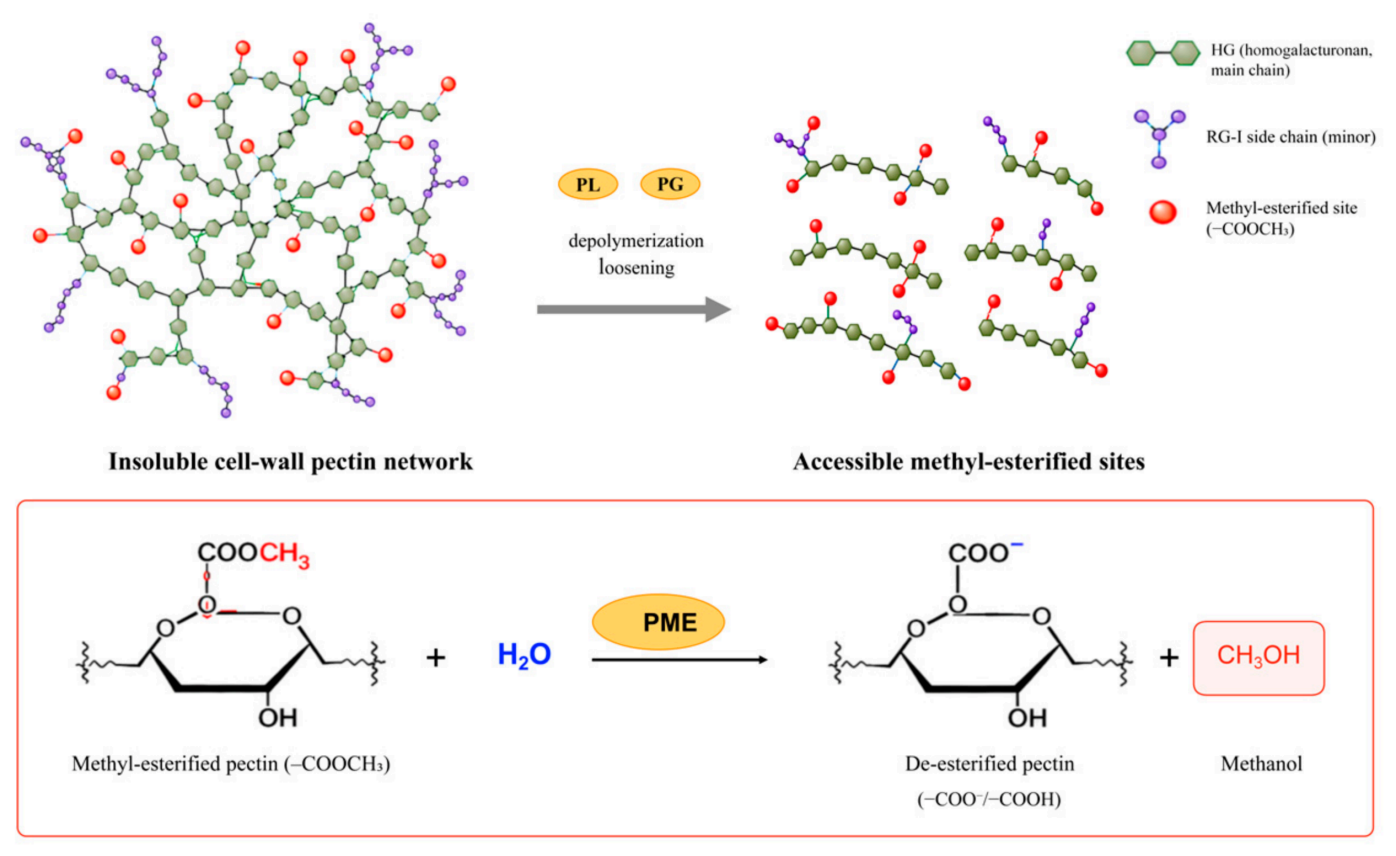
Schematic illustration of PME-mediated methanol formation from pectin.

However, the high content of pectin in kiwifruit poses significant technical challenges for wine production, as the abundant pectin substances increase the viscosity of the juice, hinder solid–liquid separation, and delay the clarification process. Therefore, pectinase treatment is usually employed to degrade pectin polysaccharides to improve processing efficiency. For instance, a recent study reported that after pectinase treatment, the juice yield reached 81.60 ± 0.47%, the clarity was 83.75 ± 0.47%, and the concentrations of various volatile compounds related to aroma also increased [11]. Despite these technical advantages, the application of pectinase may also bring potential safety concerns. Commercial pectinase preparations generally contain multiple pectinolytic activities, including pectin methylesterase (PME), polygalacturonase (PG), and pectin lyase (PL). As shown in Figure 1, PG and PL mainly depolymerize and loosen the insoluble cell-wall pectin network in kiwifruit pulp, thereby increasing the accessibility of methyl-esterified sites in the homogalacturonan region. PME subsequently catalyzes the hydrolysis of these methyl ester groups, converting methyl-esterified pectin into de-esterified pectin and releasing methanol [12]. Methanol, when metabolized in the human body, is converted into formic acid and can cause severe metabolic acidosis and selective retinal and optic nerve damage; acute intake of approximately 10 milliliters of concentrated methanol may lead to permanent blindness, while approximately 30 milliliters may be fatal [13]. Therefore, excessive pectinase treatment or insufficient control may promote the accumulation of methanol in fruit matrices rich in pectin (such as kiwifruit). According to the International Organisation of Vine and Wine (OIV), the maximum methanol concentrations are 250 mg/L for white and rosé wines and 400 mg/L for red wines; however, internationally harmonized methanol limits and systematic mitigation strategies specifically for high-pectin non-grape fruit wines remain comparatively limited [14].

Among the processing variables involved in kiwifruit wine production, pectinase dosage, fermentation temperature, and pre-fermentation clarification are particularly relevant to methanol control because they affect different stages of pectin conversion and alcoholic fermentation. Commercial pectinase preparations usually contain multiple pectinolytic activities, and inappropriate enzyme dosage may therefore enhance the availability of methanol precursors [15]. Fermentation temperature affects both yeast metabolism and the activity of pectin-related enzymes, thereby influencing alcohol production and methanol formation [16]. Clarification before fermentation can remove suspended solids, cell-wall fragments, and part of the insoluble pectin fraction, but excessive clarification may also alter the availability of nutrients and soluble pectin substrates in the fermentation system [17]. Therefore, these processing factors should be considered together rather than optimized independently.

Previous studies on fruit wine processing have mainly focused on improving fermentation efficiency, clarification, or aroma development, whereas fewer studies have integrated methanol mitigation with fermentation performance and quality retention [18,19]. For high-pectin fruit wines such as kiwifruit wine, an effective process should reduce methanol accumulation while maintaining adequate alcohol production and desirable aroma characteristics. A systematic evaluation of methanol content, fermentation indices, physicochemical properties, volatile aroma profile, and sensory quality is therefore needed to establish a quality-preserving low-methanol process.

Therefore, the present study aimed to evaluate the effects of pectinase dosage, pre-fermentation clarification, and fermentation temperature on methanol formation and fermentation performance in kiwifruit wine, and to establish a processing strategy capable of reducing methanol accumulation while maintaining adequate ethanol production and overall product quality. The selected process-regulation strategy was further evaluated in terms of physicochemical properties, volatile aroma composition, and sensory characteristics. These findings are expected to provide a mechanistic basis for researchers investigating methanol formation in high-pectin fruit wines, practical guidance for producers seeking safer and quality-preserving fermentation strategies, and scientific support for the development of kiwifruit wines with improved safety and consumer acceptability.

## 2. Methods

### 2.1. Materials and Yeast Strain

Fresh ‘Donghong’ kiwifruit (*Actinidia chinensis* cv. ‘Donghong’; Actinidiaceae) was harvested in 2025 from Furong Town, Yongshun County, Xiangxi Tujia and Miao Autonomous Prefecture, Hunan Province, China (approximately 28°45′ N, 109°56′ E). The production area has a humid mid-subtropical mountainous climate, and the 2025 growing season was generally warmer and drier than the long-term regional average.

Commercial pectinase (50,000 U/g) was obtained from Shanghai Yuanye Biotechnology Co., Ltd. (Shanghai, China). All chemical reagents were of analytical grade from Sinopharm Chemical Reagent Co., Ltd. (Shanghai, China). A low-methanol-producing strain, *Saccharomyces cerevisiae* X11, previously isolated from naturally fermented kiwifruit and preserved at the China General Microbiological Culture Collection Center (CGMCC No. 38990), was used for fermentation.

The primary seed medium was YPD liquid medium, consisting of 1.0% yeast extract, 2.0% glucose, and 2.0% peptone in distilled water, and was sterilized by autoclaving at 121 °C for 20 min before use. The secondary seed medium was prepared using ‘Donghong’ kiwifruit puree as the basal matrix. The puree was adjusted to pH 3.5 and heat-treated at 63 °C for 30 min before use.

### 2.2. Kiwifruit Wine Fermentation

**Figure 2:**
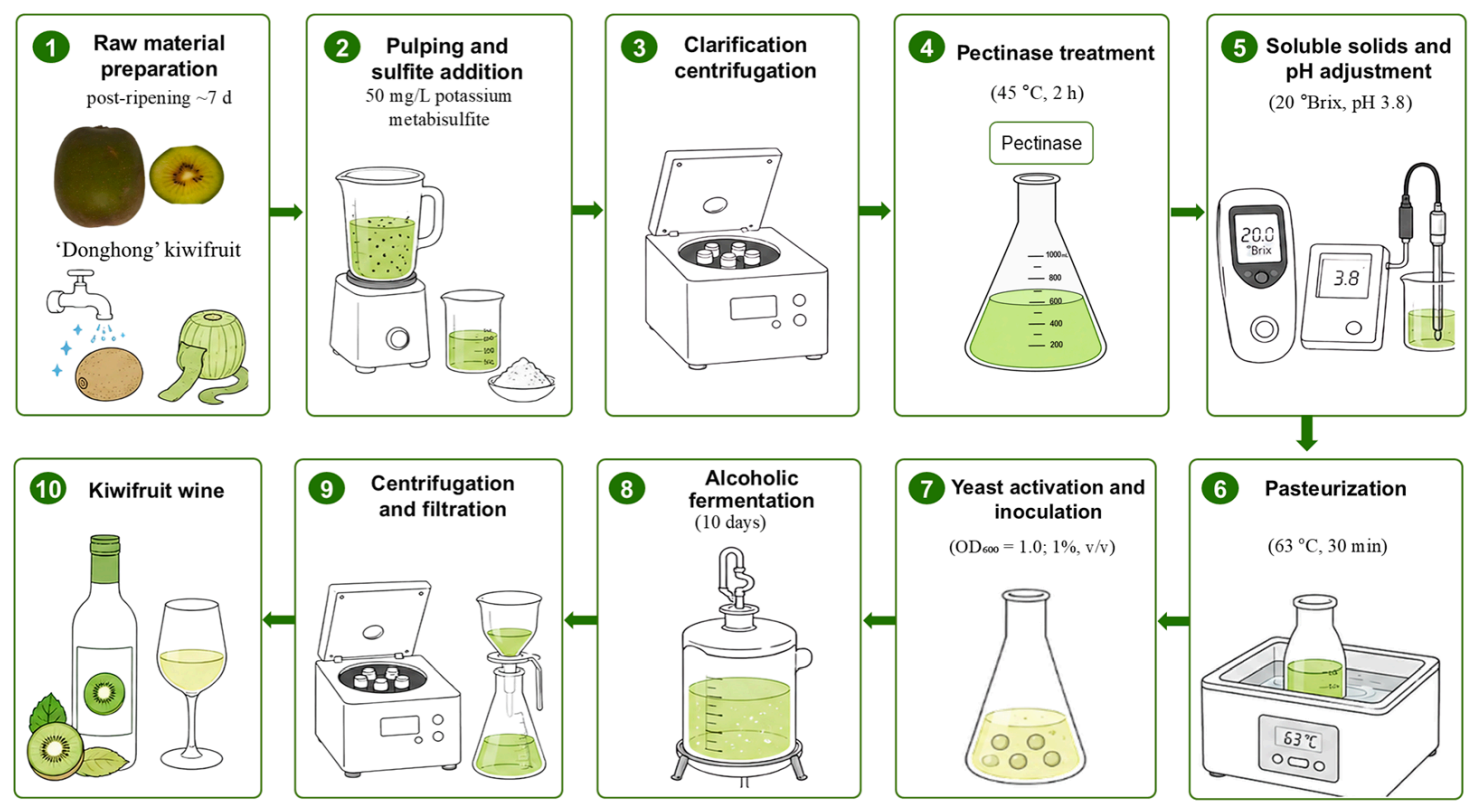
Kiwifruit wine production process.

The preparation procedure for kiwifruit wine is illustrated in Figure 2. Briefly, the kiwifruits were subjected to post-ripening in a cool, ventilated room for approximately 7 days at 20–25 °C, under natural indoor light and without direct sunlight exposure. Relative humidity was not strictly controlled but was maintained under normal indoor conditions. Fruit ripening was checked every other day according to firmness. Firm fruits were retained for further ripening, whereas moldy or decayed fruits were discarded. Fruits exhibiting slight elasticity and deformation upon gentle pressing were considered sufficiently ripened and were subsequently washed and peeled.

The peeled fruits were homogenized using an L18-P363 high-speed blender (Joyoung Co., Ltd., Jinan, China). Potassium metabisulfite was immediately added to the resulting pulp at 50 mg/L to limit oxidation and microbial contamination. The total soluble solids (TSS) were measured using a PAL-1 digital refractometer (ATAGO Co., Ltd., Tokyo, Japan), after which the pulp was stored frozen until further use.

After thawing, the kiwifruit pulp was subjected to clarification centrifugation before enzymatic treatment. For treatments involving centrifugation, the pulp was centrifuged at the designated speed to remove most of the insoluble pulp particles. The clarified pulp was then supplemented with commercial pectinase and mixed thoroughly. The pectinase preparation had a declared activity of 50,000 U/g, and the enzyme dosage was expressed as the corresponding total enzymatic activity where appropriate. Enzymatic hydrolysis was carried out in an LC-WB-6 thermostatic water bath (Shanghai Lichen Instrument Technology Co., Ltd., Shanghai, China) at 45 °C for 2 h. During this treatment, pectinolytic enzymes hydrolyzed pectic polysaccharides in the fruit matrix, promoting pectin depolymerization and facilitating subsequent clarification and solid–liquid separation.

Following enzymatic treatment, sucrose was gradually added to the kiwifruit must under continuous mixing. TSS was repeatedly monitored using the PAL-1 refractometer until a final value of 20 °Brix was reached. The pH was subsequently adjusted to 3.8 by gradual addition of sodium carbonate under continuous stirring and monitored using an H28 pH meter (Mettler-Toledo Instruments Co., Ltd., Shanghai, China). The adjusted must was then pasteurized in a thermostatic water bath at 63 °C for 30 min and cooled before yeast inoculation.

For inoculum preparation, the yeast strain was purified by streaking two to three successive times. A single colony was inoculated into the primary seed medium and cultured at 28 °C and 160 rpm for 12 h in an HCY-DA temperature-controlled shaking incubator (Taicang Haocheng Experimental Instrument Manufacturing Co., Ltd., Suzhou, China). The primary culture was subsequently transferred to the secondary seed medium and incubated under the same conditions for an additional 24 h. After secondary cultivation, the yeast suspension was diluted with sterile seed medium to an OD_600_ of 1.0, as measured at 600 nm using a UV-1800 spectrophotometer (Shimadzu, Japan). The standardized yeast suspension was then inoculated into the prepared kiwifruit must at 1% (*v*/*v*).

For each fermentation replicate, 200 g of the prepared kiwifruit must was transferred into a fermentation vessel and incubated in a temperature-controlled incubator at the temperature specified for each experimental treatment for 10 days. Fermentation progress was monitored by recording sample weight loss during the fermentation period.

At the end of alcoholic fermentation, the samples were centrifuged at 5000 rpm for 10 min using an HC-2517 high-speed centrifuge (Zhongjia Branch, Anhui USTC Zonkia Scientific Instruments Co., Ltd., Hefei, China). The supernatant was collected as the kiwifruit wine sample and stored at −20 °C until subsequent physicochemical and instrumental analyses.

### 2.3. Screening and Selection of Fermentation Conditions

The effects of fermentation temperature, pectinase dosage, and clarification centrifugation speed on methanol formation and ethanol production were investigated.

Fermentation temperatures were set at 19, 21, 23, 25, and 27 °C. Pectinase dosages ranged from 0.02 to 0.10 g/L. Clarification centrifugation was performed at 0, 2500, 5000, 7500, and 10,000 r/min for 3 min.

A control group (CK) was prepared under conventional fermentation conditions (25 °C, pectinase dosage of 0.10 g/L, and no pre-fermentation centrifugation). The processed treatment was selected based on methanol reduction efficiency while maintaining ethanol production.

Based on the results of single-factor experiments, three representative levels for each factor were selected for an L9(3^3^) orthogonal design, as shown in Table 1. Triplicates were conducted for each treatment.

### 2.4. Determination of Physicochemical Characteristics

Glucose, ethanol, and organic acids were analyzed using a Waters 2695 high-performance liquid chromatography (HPLC) system equipped with a Waters 2414 refractive index detector (RID; Waters Corporation, Milford, MA, USA). Separation was performed on an Aminex HPX-87H column (300 × 7.8 mm; Bio-Rad Laboratories, Hercules, CA, USA) maintained at 55 °C. The RID temperature was maintained at 40 °C. A 5 mmol/L H_2_SO_4_ solution was used as the mobile phase under isocratic conditions at a flow rate of 0.5 mL/min. The injection volume was 10 μL. Prior to injection, samples were filtered through a 0.22 μm polyethersulfone (PES) membrane filter. Quantification was performed using the external standard method based on calibration curves prepared from authentic standards. Because an RID was used for detection, no detection wavelength was required.

The pH was measured using an H28 pH meter (Mettler Toledo, Shanghai, China). Total acidity was determined by acid–base titration and expressed as citric acid equivalents.

Total phenolic content (TPC) was determined using a modified Folin–Ciocalteu method [20]. Gallic acid (0–150 mg/L) was used for calibration. The sample was reacted with Folin–Ciocalteu reagent and 7.5% Na_2_CO_3_, incubated in the dark, and the absorbance was measured at 765 nm using a UV-1800 UV–Vis spectrophotometer (Shimadzu, Kyoto, Japan). All measurements were performed in triplicate, and TPC was expressed as mg gallic acid equivalents per liter of wine (mg GAE/L).

Total flavonoid content (TFC) was determined using a modified NaNO_2_–AlCl_3_–NaOH colorimetric method [21]. Rutin (0–150 mg/L) was used for calibration. The sample was reacted sequentially with 30% methanol, NaNO_2_, AlCl_3_, and NaOH, and the absorbance was measured at 510 nm using a UV-1800 UV–Vis spectrophotometer (Shimadzu, Kyoto, Japan). All measurements were performed in triplicate, and TFC was expressed as mg rutin equivalents per liter of wine (mg RE/L).

### 2.5. Determination of Methanol Content

Methanol concentration was determined by headspace gas chromatography using a GC-2030 gas chromatograph (Shimadzu, Kyoto, Japan) equipped with a flame ionization detector. Briefly, 1 mL of the centrifuged wine supernatant was sealed in a headspace vial and equilibrated for 3 min. Separation was performed on a DB-WAX capillary column (30 m × 0.53 mm × 1.00 μm) at 40 °C, with helium as the carrier gas and a split ratio of 20:1. The detector temperature was set at 230 °C. Methanol was identified by retention time and quantified using an external calibration curve.

### 2.6. Analysis of Volatile Aroma Compounds

Volatile aroma compounds were analyzed by headspace solid-phase microextraction coupled with gas chromatography–mass spectrometry (HS-SPME-GC-MS): 4.5 mL of filtered wine sample was transferred into a 20 mL headspace vial containing 2 g of NaCl and 5 μL of 3-octanol as the internal standard. Analysis was performed using an Agilent 7890A gas chromatograph coupled to a 5975C mass spectrometer (Agilent Technologies, Santa Clara, CA, USA) and equipped with a DB-17MS capillary column (30 m × 0.25 mm × 0.25 μm). The oven temperature was held at 40 °C for 5 min, increased to 70 °C at 2 °C/min and held for 2 min, then raised to 120 °C at 3 °C/min, to 150 °C at 5 °C/min, and finally to 220 °C at 10 °C/min with a 2 min hold. The injector temperature was 250 °C. High-purity helium was used as the carrier gas at a flow rate of 1.0 mL/min. Mass spectrometric detection was performed in the electron ionization mode, with the ion source and transfer line maintained at 230 and 280 °C, respectively, over a scan range of *m*/*z* 30–300. Volatile compounds were tentatively identified by comparison with the NIST 19 Mass Spectral Library (NIST Standard Reference Database 1A) and relatively quantified using peak-area normalization combined with the internal standard method. Because volatile compounds were relatively quantified using the internal standard method rather than absolutely quantified with authentic standards for each compound, the aroma analysis focused on relative profile differences among treatments.

### 2.7. Sensory Evaluation

Sensory evaluation was conducted by a panel of 10 assessors (5 males and 5 females), aged 22–28 years, all with postgraduate-level educational backgrounds. All assessors had substantial previous experience in wine tasting and were familiar with the basic sensory characteristics of alcoholic beverages. Before the formal evaluation, the sensory attributes, scoring criteria, and evaluation procedure were explained to all panelists to ensure a consistent understanding of the 5-point scale. All participants were fully informed of the experimental procedure and signed written informed consent forms before participation.

Prior to sensory evaluation, the methanol concentration of each wine sample was analytically determined. To minimize potential methanol and ethanol exposure, only a small volume of each sample was provided for tasting, and panelists were instructed to expectorate the samples after oral evaluation rather than swallow them. All samples used for sensory evaluation had been analytically confirmed to be below the relevant methanol safety reference limit before tasting. The evaluation was conducted in a spacious, well-lit room free from noticeable odors and other environmental interference. Five kiwifruit wine samples were assigned random three-digit codes and presented in randomized order. Panelists first evaluated appearance and aroma, followed by taste and aftertaste, and independently scored each predefined sensory attribute on a 5-point scale. Purified water was provided for palate cleansing between samples, and communication among panelists was not permitted during evaluation. The mean score of the 10 assessors was used for subsequent analysis.

### 2.8. Statistical Analysis

All experiments were performed in triplicate, and results were expressed as mean ± standard deviation. Statistical significance was determined using one-way analysis of variance (ANOVA) followed by Duncan’s multiple range test (*p* < 0.05). Principal component analysis (PCA), heatmap clustering, and graphical visualization were performed using OriginPro 2025 and R software 4.4.1.

## 3. Results

### 3.1. Effects of Fermentation Conditions on Methanol Formation and Ethanol Production

**Figure 3:**
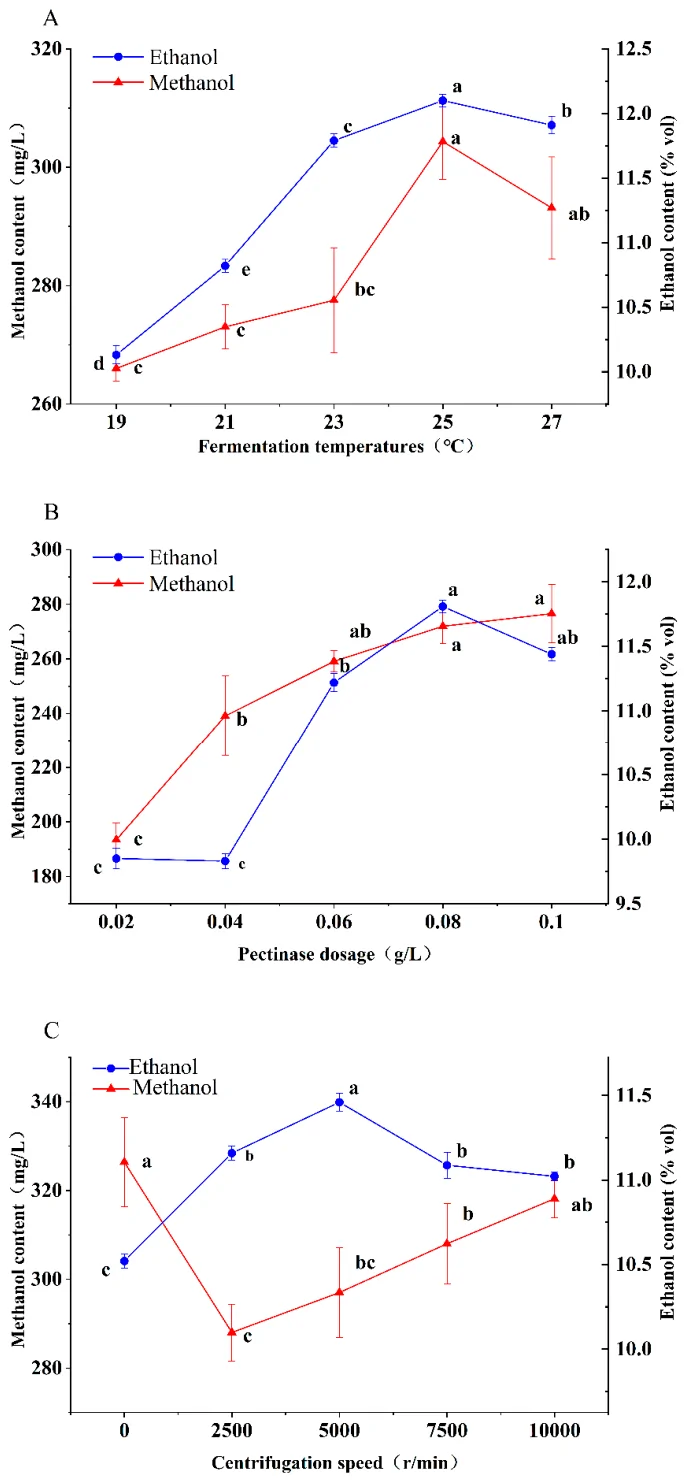
Single-factor effects of fermentation conditions on methanol and ethanol contents in kiwifruit wine. (**A**) Fermentation temperature; (**B**) pectinase dosage; (**C**) centrifugation speed. Data are presented as mean ± standard deviation (*n* = 3). Different letters indicate significant differences among treatments (*p* < 0.05).

The effects of fermentation temperature, pectinase dosage, and clarification centrifugation speed on methanol formation and ethanol production were investigated, and the results are shown in Figure 3. Because different single-factor experiments were conducted under their respective baseline conditions, the results were primarily used to assess the variation trends attributable to individual factors.

Fermentation temperature showed a clear influence on methanol formation and ethanol production (Figure 3A). Methanol content gradually increased from 266.01 ± 2.19 mg/L at 19 °C to 304.34 ± 6.43 mg/L at 25 °C, followed by a slight decrease at 27 °C to 293.14 ± 8.60 mg/L. Ethanol production exhibited a similar trend, peaking at 25 °C and then decreasing slightly. The increase in methanol content with rising temperature may be associated with enhanced pectin solubilization and increased activity of pectin-related enzymes, thereby promoting the release and conversion of methanol precursors [22]. The slight decline in ethanol production at 27 °C may indicate that excessively high temperatures were unfavorable for sustained alcoholic fermentation [23]. Considering the balance between methanol reduction and fermentation performance, lower-temperature conditions were selected for further optimization.

Pectinase dosage exhibited a stronger effect on methanol accumulation than the other investigated factors (Figure 3B). Methanol content increased continuously with increasing pectinase dosage and reached the highest level of 276.58 ± 10.73 mg/L at 0.10 g/L, whereas ethanol production increased up to 0.08 g/L and then decreased slightly. The increase in methanol content may be attributed to enhanced pectin degradation and greater release of methyl ester groups during enzymatic hydrolysis [24]. Although a higher pectinase dosage could improve pulp liquefaction and substrate availability, its contribution to ethanol production was limited at excessive levels, while increasing the risk of methanol formation [25]. Therefore, pectinase dosages of 0.02–0.06 g/L were selected for further optimization.

Interestingly, methanol content did not decrease continuously with increasing centrifugation speed, as might be expected from the enhanced removal of pectin-rich suspended solids. Instead, it reached a minimum at 2500 r/min and then gradually increased at higher speeds, as shown in Figure 3C. Moderate centrifugation may remove suspended particles containing cell-wall materials and pectinaceous substances, thereby reducing the amount of pectin substrate available for methanol formation [26]. However, because the total sample mass was standardized across treatments, the greater removal of insoluble solids at higher centrifugation speeds may have increased the relative proportion of soluble pectin in the fermentation matrix. This could enhance substrate accessibility to pectin-related enzymes and partly account for the subsequent increase in methanol formation [27].

**Figure 4:**
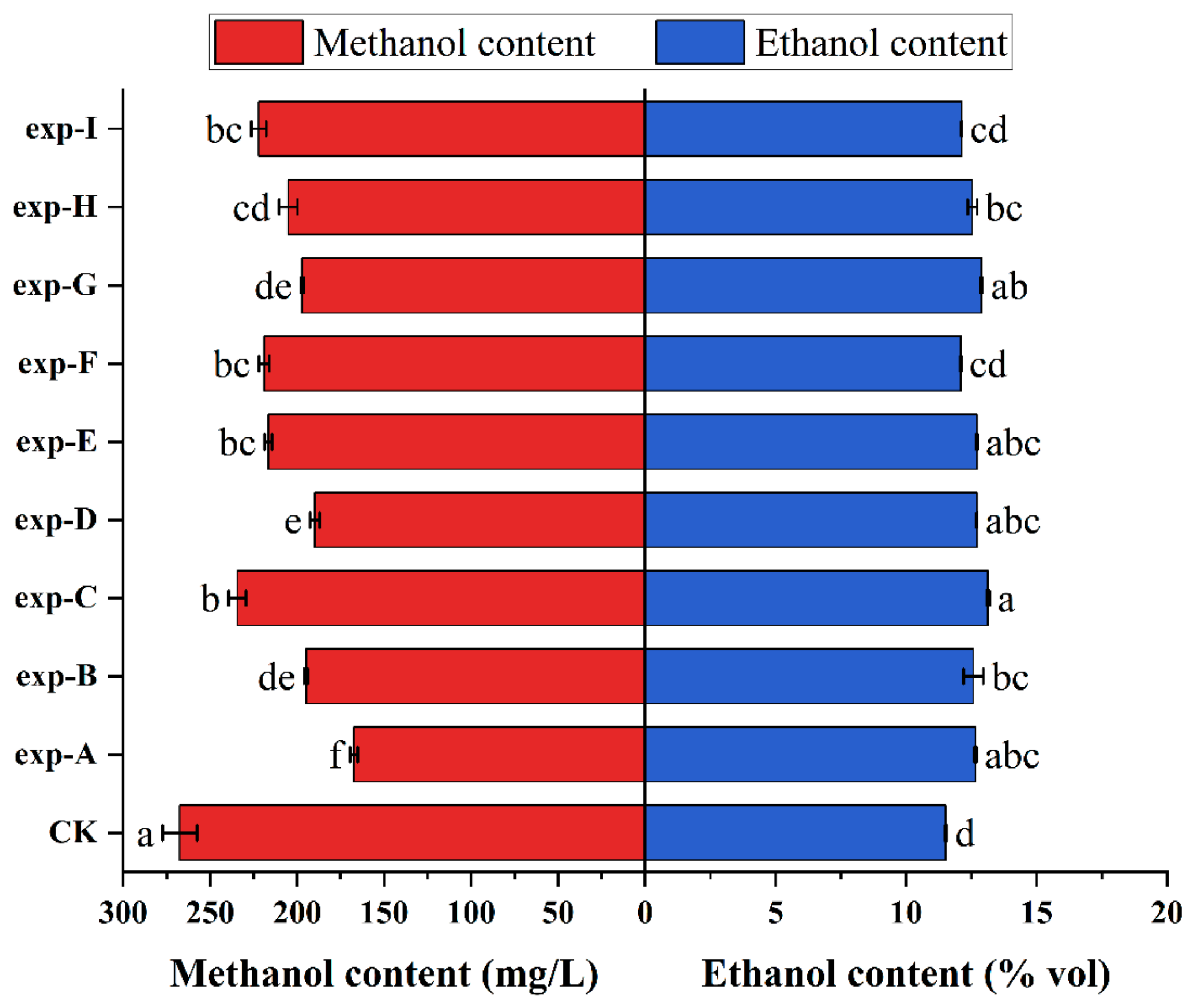
Effects of orthogonal treatments on methanol formation and ethanol production in kiwifruit wine. Different lowercase letters indicate significant differences among treatments according to Duncan’s multiple range test (*p* < 0.05).

Based on the single-factor results, the ranges of fermentation temperature (19–23 °C), pectinase dosage (0.02–0.06 g/L), and centrifugation speed (2500–7500 r/min) were selected for the L9 (3^3^) orthogonal design. Nine orthogonal runs were generated according to different combinations of the three factors (Figure 4). Among these treatments, exp-A exhibited the lowest methanol content (167.45 ± 2.20 mg/L) while maintaining a relatively high ethanol content (12.66 ± 0.04% vol). Compared with the CK group (267.45 ± 9.91 mg/L methanol and 11.92 ± 0.02% vol ethanol), exp-A reduced methanol formation by 37.39% and increased ethanol content, indicating that the selected fermentation conditions effectively mitigated methanol generation while maintaining fermentation performance. Therefore, exp-A was selected as the optimal fermentation condition.

The analysis of variance results are summarized in Table 2. Pectinase dosage was the only significant factor affecting methanol formation (*p* < 0.05), whereas fermentation temperature and centrifugation speed had no significant effect (*p* > 0.05). In contrast, none of the investigated factors significantly affected ethanol production (*p* > 0.05). These results indicate that process regulation primarily influenced methanol accumulation, with limited effects on yeast alcohol fermentation performance.

### 3.2. Physicochemical Characteristics of Kiwifruit Wines Produced Under Different Fermentation Treatments

**Figure 5:**
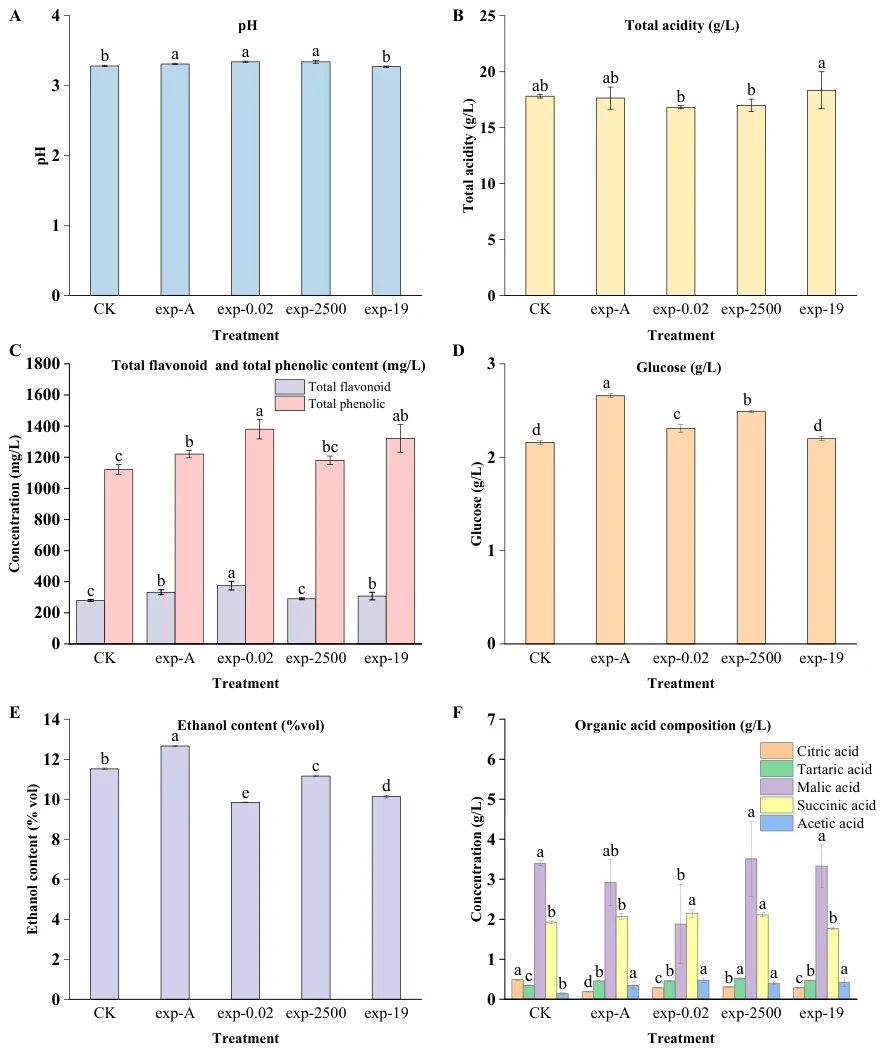
Physicochemical characteristics of kiwifruit wines produced under different fermentation treatments. (**A**) pH; (**B**) total acidity; (**C**) total flavonoid and total phenolic contents; (**D**) glucose content; (**E**) ethanol content; and (**F**) organic acid composition. CK: control treatment; exp-A: optimized treatment obtained from the orthogonal experiment; exp-0.02: lowest-methanol treatment in the pectinase dosage experiment; exp-2500: lowest-methanol treatment in the centrifugation experiment; exp-19: lowest-methanol treatment in the fermentation temperature experiment. Data are expressed as mean ± standard deviation (*n* = 3). Different lowercase letters indicate significant differences among treatments according to Duncan’s multiple range test (*p* < 0.05).

Based on the single-factor and orthogonal experiments, five representative wines were selected for physicochemical evaluation, including CK, three single-factor treatments (exp-0.02, exp-2500, and exp-19), and the optimized treatment exp-A. The physicochemical characteristics differed among treatments, but all samples maintained the basic compositional features of kiwifruit wine as shown in Figure 5.

The pH values varied within a narrow range of 3.27–3.34, indicating that the different processing conditions had only a limited influence on the overall acid–base environment. Exp-A, exp-0.02, and exp-2500 showed slightly higher pH values than CK and exp-19, whereas exp-19 exhibited the highest total acidity. The incomplete correspondence between pH and total acidity may be attributed to differences in organic acid composition, dissociation behavior, and buffering capacity. Total flavonoid and total phenolic contents were higher in the treated wines than in CK, with the highest values observed in exp-0.02. Exp-A also retained significantly higher levels of these compounds than CK, suggesting that the processed process did not adversely affect the retention of phenolic-related components.

Residual glucose and ethanol contents showed clear treatment-dependent differences. Exp-A exhibited the highest ethanol content and residual glucose levels, indicating that the combined optimized conditions supported efficient alcoholic fermentation while maintaining a relatively high availability of fermentable sugars. The lower fermentation temperature may have moderated glucose consumption, allowing substantial ethanol accumulation without complete depletion of residual glucose. In addition, the combined effects of pectinase dosage and clarification treatment may have altered sugar release and substrate accessibility during fermentation. In contrast, exp-0.02 and exp-19 showed relatively low ethanol contents, suggesting that adjusting a single processing factor was less effective at maintaining fermentation performance. The combined regulation used in exp-A therefore appeared to provide a more favorable balance between substrate availability and ethanol production.

The organic acid profile varied markedly among the fermentation treatments. Malic acid was the predominant organic acid in all samples and therefore the major contributor to the sharp and refreshing acidity of kiwifruit wine [28]. Citric acid generally contributes a clean and bright sourness, whereas tartaric acid helps maintain wine acidity and structure [29]. Succinic acid, a secondary metabolite of alcoholic fermentation, may contribute not only to sourness but also to slight bitterness and saltiness, while excessive acetic acid can increase volatile acidity and impair sensory acceptability [30]. In this study, exp-A showed the lowest citric acid content, which may reflect greater citric acid utilization under the combined optimized conditions and is consistent with its relatively high ethanol production. Malic and succinic acid contents also differed among treatments, suggesting that fermentation temperature, pectinase dosage, and clarification intensity altered substrate availability and redirected organic acid metabolism [31]. Although acetic acid increased in the treated samples, all concentrations remained below 0.5 g/L, indicating limited accumulation of volatile acidity and a low risk of pronounced vinegar-like off-flavor [32].

### 3.3. Volatile Aroma Characteristics of Kiwifruit Wines Under Different Fermentation Treatments

To evaluate the influence of process regulation on the flavor quality of kiwifruit wine, the volatile aroma profiles of different treatment groups were analyzed using a hierarchical clustering heatmap and principal component analysis (PCA). The results indicated that fermentation temperature, pectinase dosage, and clarification centrifugation not only affected methanol formation but also contributed to variations in volatile aroma composition.

**Figure 6:**
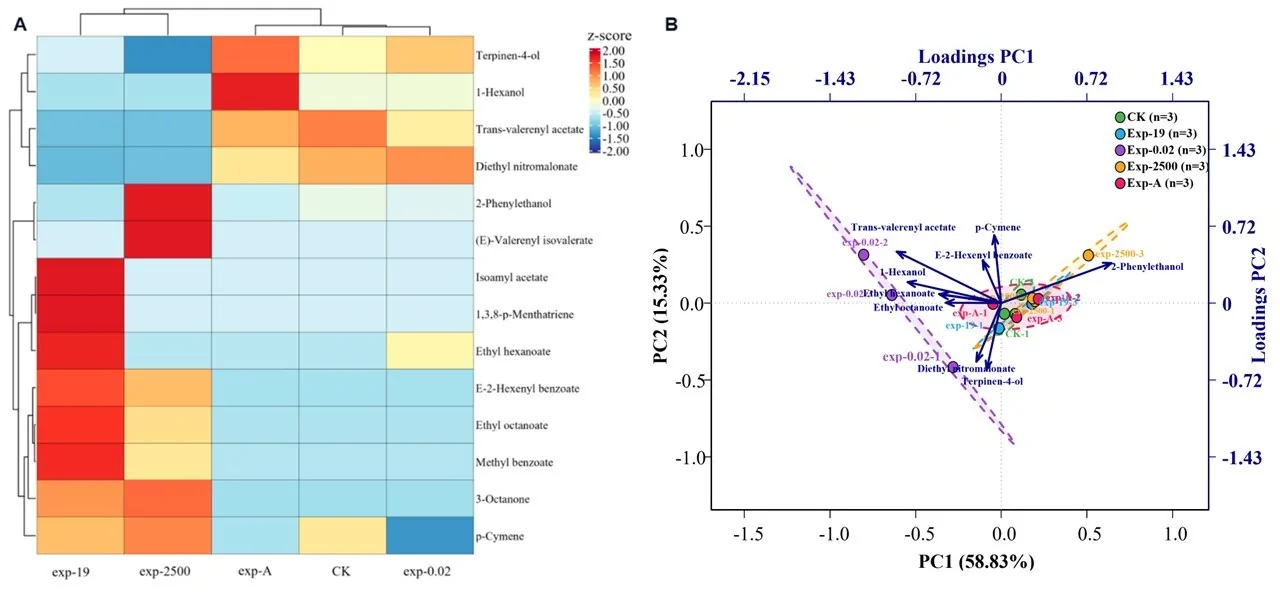
Volatile aroma profiles of kiwifruit wines under different fermentation treatments. (**A**) Hierarchical clustering heatmap of representative volatile compounds; (**B**) PCA score plot based on volatile profiles.

The clustering heatmap revealed differences in the distribution patterns of characteristic volatile compounds among the different treatments, as demonstrated in Figure 6A. CK, exp-0.02, and exp-A were grouped in the same cluster, whereas exp-2500 and exp-19 exhibited relatively similar aroma profiles, suggesting that the treatment altered the volatile composition to some extent but did not result in complete separation among samples. Among them, exp-A showed relatively higher levels of 1-hexanol and terpinen-4-ol. 1-Hexanol is commonly associated with green and herbaceous notes, while terpinen-4-ol contributes herbal, woody, and spicy characteristics, indicating that the selected process-regulation strategy may enhance certain green and botanical aroma attributes while maintaining the original aroma characteristics of kiwifruit wine [33]. In exp-19, several ester compounds, including ethyl hexanoate, isoamyl acetate, methyl benzoate, and ethyl octanoate, exhibited relatively high abundances, suggesting that low-temperature fermentation may promote the accumulation of fruity and fermented aroma compounds [34]. The exp-2500 group showed higher relative abundances of 2-phenylethanol and certain ester compounds, which may contribute to floral, sweet, and fruity aroma characteristics [35].

PCA was further performed to evaluate the overall differences in volatile aroma profiles among the samples in Figure 6B. The first two principal components (PC1 and PC2) accounted for 58.83% and 15.33% of the total variance, respectively, with a cumulative contribution rate of 74.16%, indicating that the PCA model effectively represented the variation in volatile compounds among different treatments. The exp-A samples were located close to CK in the PCA score plot, indicating that the selected fermentation process effectively reduced methanol formation while maintaining a volatile aroma profile similar to that of the control group. The partial overlap between exp-2500, CK, and exp-A indicated that clarification centrifugation had a relatively limited influence on the overall aroma structure. In contrast, exp-19 showed partial separation from CK and exp-A along the PC1 axis, suggesting that fermentation temperature played an important role in regulating aroma composition. The relatively scattered distribution of exp-0.02 samples indicated that reducing pectinase dosage alone might affect the stability of aroma formation.

The loading analysis showed that alcohols and esters were the main volatile compounds contributing to the differentiation of aroma profiles. Specifically, 1-hexanol, terpinen-4-ol, ethyl octanoate, ethyl hexanoate, and E-2-hexenyl benzoate exhibited relatively high contributions to the principal components, suggesting that these compounds were important contributors to aroma variation among different treatments.

### 3.4. Sensory Evaluation of Kiwifruit Wines Under Different Fermentation Treatments

**Figure 7:**
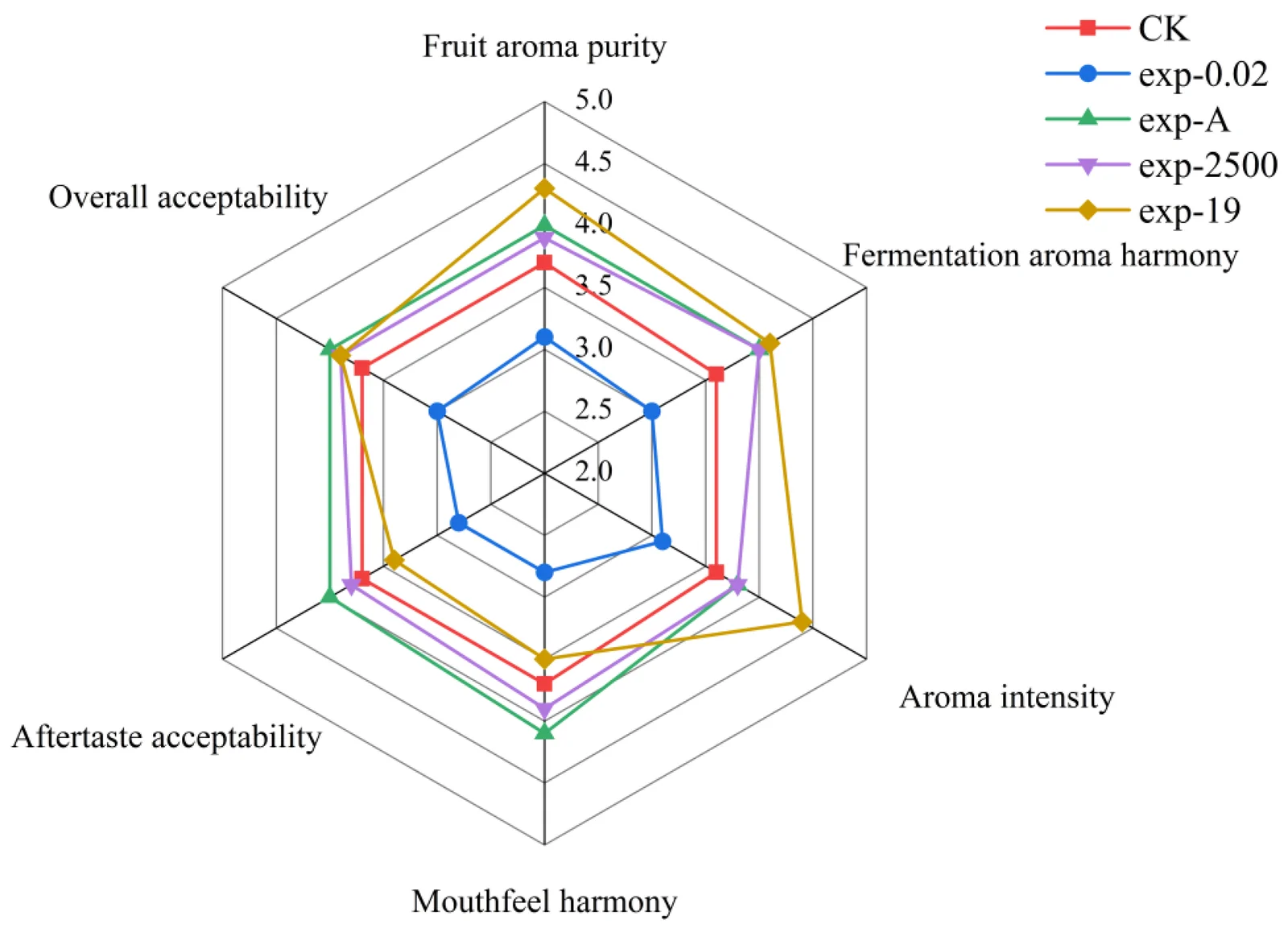
Sensory evaluation radar plot of kiwifruit wines under different fermentation treatments.

Sensory evaluation was conducted to further determine whether process regulation influenced the perceived aroma and taste quality of kiwifruit wine. As shown in Figure 7, the five treatments differed in fruit aroma purity, fermentation aroma harmony, aroma intensity, mouthfeel harmony, aftertaste acceptability, and overall acceptability. These sensory attributes reflected not only the perception of volatile aroma compounds but also the integrated effects of ethanol, organic acids, phenolic compounds, residual sugars, and the overall balance of the wine matrix [36,37,38].

Among the treatments, exp-19 obtained the highest scores for fruit aroma purity and aroma intensity, suggesting that low-temperature fermentation favored the preservation or formation of aroma attributes perceived as fresh and fruity. This trend was generally consistent with the heatmap results, in which exp-19 showed relatively higher abundances of several ester- and terpene-related compounds [39]. However, exp-19 showed lower scores for mouthfeel harmony and aftertaste acceptability, indicating that stronger aroma intensity did not necessarily result in better overall sensory quality. This may be related to its higher acidity and less balanced taste structure [40].

Exp-A showed a more balanced sensory profile, with relatively high scores for mouthfeel harmony, aftertaste acceptability, and overall acceptability. Although exp-A did not show the strongest intensity in all aroma-related attributes, its sensory profile was more coordinated than those of the single-factor treatments. This result was consistent with the PCA analysis, in which exp-A was located close to CK, suggesting that the low-methanol process maintained the overall aroma structure of the control wine while improving sensory balance.

In contrast, exp-0.02 received the lowest scores for most sensory attributes, especially fermentation aroma harmony, mouthfeel harmony, and aftertaste acceptability. This result suggested that reducing pectinase dosage alone may affect the release of aroma-related compounds and the coordination of fermentation-derived flavor [41]. Overall, the sensory evaluation confirmed that methanol mitigation should be considered together with flavor quality. The exp-A treatment reduced methanol formation while maintaining or improving the overall sensory acceptability of kiwifruit wine.

## 4. Discussion

Methanol formation in kiwifruit wine was closely related to pectin degradation and pectinase-associated enzymatic reactions. In this study, methanol content increased with increasing pectinase dosage, and pectinase dosage was the only significant factor affecting methanol formation in the orthogonal experiment. This trend can be explained by the fact that commercial pectinase preparations usually contain multiple pectinolytic activities, including polygalacturonase, pectin lyase, and pectin methylesterase. Among them, pectin methylesterase directly hydrolyzes methyl ester groups in pectin and releases methanol, whereas polygalacturonase and pectin lyase may depolymerize the pectin network and increase the accessibility of methyl-esterified sites [14]. Therefore, excessive pectinase addition may enhance pectin demethylation and promote methanol accumulation.

The selected treatment reduced methanol mainly through the combined regulation of pectinase dosage, fermentation temperature, and clarification centrifugation. A lower pectinase dosage likely limited PME-related demethylation, while fermentation at 19 °C may have reduced pectin solubilization and enzyme activity compared with higher-temperature treatments. Moderate centrifugation at 2500 r/min may have removed part of the suspended cell-wall fragments and pectin-rich particles before fermentation, thereby decreasing the availability of methanol precursors. However, excessive clarification did not further reduce methanol, possibly because it changed the relative proportion of soluble pectin and increased the accessibility of residual pectin substrates. These results indicate that methanol reduction was achieved by limiting both pectin substrate availability and enzymatic demethylation conditions.

Importantly, this low-methanol process did not negatively affect fermentation performance or aroma quality. Methanol is mainly derived from pectin methyl ester groups, whereas ethanol is produced from fermentable sugars by yeast metabolism. Therefore, controlling pectin degradation can reduce methanol formation without necessarily inhibiting alcoholic fermentation. In addition, low-temperature fermentation may help retain volatile aroma compounds, while an appropriate pectinase dosage can still promote moderate cell-wall degradation and the release of aroma-related precursors [42]. This may explain why exp-A maintained a volatile aroma profile close to CK in the PCA analysis while achieving a marked reduction in methanol content.

The sensory results further showed that the quality of kiwifruit wine was determined by the overall balance between aroma and taste rather than by aroma intensity alone. Although exp-19 showed higher fruit aroma purity and aroma intensity, its lower mouthfeel harmony and aftertaste acceptability may be associated with higher acidity and a less balanced taste structure. In contrast, exp-A showed better overall acceptability, suggesting that the improved process maintained the coordination among volatile aroma compounds, ethanol, organic acids, phenolic compounds, residual sugars, and other matrix components [40]. Overall, the reduction in methanol and the preservation of aroma quality in exp-A can be attributed to the coordinated control of pectin degradation, fermentation temperature, and clarification intensity, providing a practical strategy for producing safer kiwifruit wine without substantial loss of sensory quality.

## 5. Conclusions

This study established a process regulation strategy for mitigating methanol formation in kiwifruit wine by coordinating pectinase dosage, fermentation temperature, and pre-fermentation clarification. Among the investigated factors, pectinase dosage showed the strongest influence on methanol accumulation, confirming that methanol control in kiwifruit wine should focus on the regulation of pectin degradation rather than on alcoholic fermentation alone.

The improved process effectively reduced methanol formation while maintaining ethanol production and the major physicochemical characteristics of kiwifruit wine. This indicates that appropriate regulation of pectin-related processing conditions can improve the safety profile of high-pectin fruit wines without substantially weakening fermentation performance.

Volatile aroma analysis and sensory evaluation further showed that this treatment maintained an aroma profile comparable to that of the control and achieved a more balanced sensory performance. These results suggest that methanol mitigation and flavor preservation can be achieved simultaneously when enzymatic treatment, clarification intensity, and fermentation temperature are properly coordinated.

Overall, this work provides a practical approach for producing safer kiwifruit wine with preserved quality attributes. It may also serve as a reference for methanol control in other high-pectin fruit wine systems. Future studies should further evaluate the storage stability, turbidity stability, microbial safety, and scale-up applicability of the process.

## Figures and Tables

**Table 1:** Factors and levels of the L9 (3^3^) orthogonal experimental design.

Treatment	Orthogonal Test Factor		
	Fermentation Temperature (°C)	Pectinase Dosage (g/L)	Centrifugation Speed (r/min)
exp-A	19	0.02	2500
exp-B	19	0.04	5000
exp-C	19	0.06	7500
exp-D	21	0.02	5000
exp-E	21	0.04	7500
exp-F	21	0.06	2500
exp-G	23	0.02	7500
exp-H	23	0.04	2500
exp-I	23	0.06	5000

**Table 2:** Analysis of variance for methanol and ethanol contents in orthogonal experiments.

Response	Factor	Sum of Squares	df	F-Value	*p*-Value	Significance
Methanol	Fermentation temperature	180.14	2	1.81	0.19	NS
	Pectinase dosage	2439.10	2	24.52	0.02	*
	Centrifugation speed	582.61	2	5.86	0.15	NS
	Error	99.47	2	—	—	—
Ethanol	Fermentation temperature	0.160	2	0.97	0.508	NS
	Pectinase dosage	0.135	2	0.82	0.549	NS
	Centrifugation speed	0.428	2	2.61	0.277	NS
	Error	0.164	2	—	—	—

NS, not significant; *, *p* < 0.05, was considered statistically significant.

## Data Availability

The data presented in this study are available upon request from the corresponding author due to university policy.
